# Tetracalcium Phosphate/Monetite/Calcium Sulfate Hemihdrate Biocement Powder Mixtures Prepared by the One-Step Synthesis for Preparation of Nanocrystalline Hydroxyapatite Biocement-Properties and In Vitro Evaluation

**DOI:** 10.3390/ma14092137

**Published:** 2021-04-22

**Authors:** Lubomir Medvecky, Maria Giretova, Radoslava Stulajterova, Lenka Luptakova, Tibor Sopcak

**Affiliations:** 1 Department of Functional and Hybrid Materials, Institute of Materials Research of SAS, Watsonova 47, 04 001 Kosice, Slovakia; mgiretova@saske.sk (M.G.); rstulajterova@saske.sk (R.S.); tsopcak@saske.sk (T.S.); 2 Department of Biology and Physiology, University of Veterinary Medicine and Pharmacy in Kosice, Komenskeho 73, 041 81 Kosice, Slovakia; lenka.luptakova@uvlf.sk

**Keywords:** calcium phosphate cement, hemihydrate calcium sulfate, microstructure, setting process, gene expression, mesenchymal stem cells

## Abstract

A modified one-step process was used to prepare tetracalcium phosphate/monetite/calcium sulfate hemihydrate powder cement mixtures (CAS). The procedure allowed the formation of monetite and calcium sulfate hemihydrate (CSH) in the form of nanoparticles. It was hypothesized that the presence of nanoCSH in small amounts enhances the in vitro bioactivity of CAS cement in relation to osteogenic gene markers in mesenchymal stem cells (MSCs). The CAS powder mixtures with 15 and 5 wt.% CSH were prepared by milling powder tetracalcium phosphate in an ethanolic solution of both orthophosphoric and sulfuric acids. The CAS cements had short setting times (around 5 min). The fast setting of the cement samples after the addition of the liquid component (water solution of NaH_2_PO_4_) was due to the partial formation of calcium sulfate dihydrate and hydroxyapatite before soaking in SBF with a small change in the original phase composition in cement powder samples after milling. Nanocrystalline hydroxyapatite biocement was produced by soaking of cement samples after setting in simulated body fluid (SBF). The fast release of calcium ions from CAS5 cement, as well as a small rise in the pH of SBF during soaking, were demonstrated. After soaking in SBF for 7 days, the final product of the cement transformation was nanocrystalline hydroxyapatite. The compressive strength of the cement samples (up to 30 MPa) after soaking in simulated body fluid (SBF) was comparable to that of bone. Real time polymerase chain reaction (RT-PCR) analysis revealed statistically significant higher gene expressions of alkaline phosphatase (ALP), osteonectin (ON) and osteopontin (OP) in cells cultured for 14 days in CAS5 extract compared to CSH-free cement. The addition of a small amount of nanoCSH (5 wt.%) to the tetracalcium phosphate (TTCP)/monetite cement mixture significantly promoted the over expression of osteogenic markers in MSCs. The prepared CAS powder mixture with its enhanced bioactivity can be used for bone defect treatment and has good potential for bone healing.

## 1. Introduction

Calcium phosphate-based cements (CPC) are used to treat bone defects because they are similar to bone hydroxyapatite and have excellent biocompatibility and osteoconduction [1,2]. One group of CPCs are self-setting cements, based on tetracalciumcalcium phosphate/monetite powder mixtures (TTCPM), which are characterized by rapid setting and a gradual transformation of calcium phosphate components to calcium deficient hydroxyapatite (HAP) after mixing with a liquid component [3,4]. The drawbacks of TTCPM biocements are a slower rate of resorption in vivo, a relatively rapid change of pH to a strong basic region after the preparation of the cement paste, and, consequently, insufficient osteoinduction after defect treatment. The increase in cement solubility or basicity of cement paste can be solved by changing the Ca/P molar ratio in the cement, the acid character of liquid component (e.g., soluble organic substances (carboxylic acids, phytic acid)) and forming porous scaffolds using porogens biodegradable biopolymers [5,6,7]. The rise in cement solubility with enhanced release of calcium from the cement improves the bioactivity of cement in relation to osteoinduction [8]. Another possibility for improvement of TTCPM bioactivity is addition of more soluble inorganic components, which actively influence cell behavior after the application of cements; from this point of view, the addition of a complex amino acid mixture or arginine-glycine-aspartate (RGD) [9,10], the preparation of TTCPM/magnesium phosphate composite cements with more soluble magnesium components [11], substitution of HAP with sulfate ions [12], and CS/CPC composites [13] represent cheap and simple examples of studied biocements. Calcium sulfate in the form of hemihydrate (CSH) has been used as a cheap, synthetic, non-toxic, and bioactive bone filler over 100 years. However, it has certain limitations, such as poor handling properties, poor mechanical properties, and a very fast resorption rate after its application to a defect site. Contrary to the above facts, a positive effect of CSH (αCSH is superior to βCSH) on the proliferation and differentiation of osteoblasts, as well as up-regulation of osteogenic gene markers (ALP, osteocalcin and osteonectin), has been demonstrated [14]. Generally, calcium sulfate is commercially added to some types of CPCs, where it is used as a soluble non-toxic porogen [15]. A fast in vivo resorption and the formation of a new cancellous bone were found in rabbits after 4 weeks from the implantation of coarse (100–150 µm size) porous composite calcium phosphate/calcium sulfate dihydrate (CSD) [16]. Similarly, the addition of CSH to HAP cement showed a marked improvement in in vivo properties, such as good biocompatibility after implantation in rat muscle and a gradual bioresorption over 12 weeks with blood vessels penetrating the material [17]. On the other hand, the ability of CSH to achieve rapid transformation in 0.1 M (NH_4_)_2_HPO_4_ solution to amorphous calcium phosphate and dicalcium phosphate with a composition close to tricalcium phosphate has been verified [18], and this result is in accordance with the results of in vivo experiments. In the case of setting time, small variations in setting time (ST) of fast setting αTCP cement with a change of H_2_SO_4_ concentration (up to 0.33 M) in a liquid component have been revealed [19], contrary to the strong effect of the liquid/powder (L/P) ratio found in CPC/CSH cement pastes, which clearly indicates the need to optimize this parameter [20]. Enhanced injectability was measured in TTCPM/CSH cement mixtures with short setting times and showed improved mechanical properties compared to brushite/CSH cements [21,22]. The TCP/CSH cement mixture showed a weak CS (about 5 MPa) with the formation of brushite and calcium sulfate dihydrate (CSD) after setting with NaH_2_PO_4_ solution in a mold [23], while, in the case of TTCPM/CSD cement (even with 50 wt.% of CSD), a rapid transformation of cement components to hydroxyapatite was identified in a short time [24]. Note that the addition of TTCPM mixture to CSH affected the nucleation and growth of CSD and also the kinetics of cement transformation. Despite an improvement in cement bioactivity, the compressive strength of CPC/CSH cements decreased when adding CSH to the cement due to the dissolution of CS [20] and from changes in the porosity of cements [25]; these facts need to be taken into account in the preparation and practical application of cements. In all the above mentioned cases, TTCPM/CSH cement mixtures were prepared in several steps via mechanical homogenization of microcrystalline powder precursors.

The aim of the work was to characterize the microstructure, phase evolution, and setting process of TTCP/monetite/CSH biocement powder mixtures prepared using a one-step synthesis, as well as the in vitro testing of the cytotoxicity of cement extracts and analysis of the proliferation of mesenchymal stem cells (MSC) cultured in cement extracts. Moreover, the effect of nanoCSH addition on TTCPM cement bioactivity was studied through analysis of specific osteogenic cell markers and gene expression (osteopontin (OP), osteonectin (ON), osteocalcin (OCN), ALP, collagen I (COLL I)) in MSCs cultured in cement extracts.

## 2. Materials and Methods

### 2.1. Preparation of Powder Cement Mixtures, Pastes and Cement Samples

Tetracalcium phosphate (Ca_4_(PO_4_)_2_O, TTCP) was prepared according to the method described in [9]. The TTCPM/nanoCSH powder cement mixtures were synthesized via an in situ reaction of TTCP with orthophosphoric acid (86% analytical grade, Merck, Darmstadt, Germany)/H_2_SO_4_ (96%, analytical grade, Merck) mixture in 80 *v*/*v* % ethanol (reaction solution) using a planetary ball mill with agate balls and a vessel for 30 min. The orthophosphoric acid was added in such an amount to have the Ca/P mole ratio in the cements close to 1.67 (designated C cement, free of CSH) and sulfuric acid was added in amounts of 5 and 15 wt.% of CSH designated CAS5 and CAS15, both acids added) in the final cements. The cement pastes were prepared by mixing the powder mixtures with 2% NaH_2_PO_4_ (as a liquid component) at a powder/liquid (P/L) ratio = 2. The samples for mechanical testing were prepared by molding the cement pastes into a cylindrical shape (6 mm *D* × 12 mm *H*). The samples were soaked in SBF solution at pH = 7.4 and 37 °C for 7 days after setting in 100% humidity at 37 °C for 10 min, which was sufficient time for the samples to not disintegrate after immersion into the solution, and following immersion in SBF.

Note that the main difference between the C cement and TTCPM cements studied in other papers is the preparation procedure, where the TTCPM cement mixtures were prepared using simple mechanical homogenization of the starting TTCP and DCPA powder phases, contrary to the in situ synthesis of the monetite from TTCP phase in C cement.

### 2.2. XRD Phase Analysis, Chemical Composition and Microstructure of Cements

The phase composition of samples were characterized by X-ray diffraction analysis (Philips X’ PertPro, Malvern Panalytical B.V., Eindhoven, The Netherlands, using Cu Kα radiation, 40 kV, 50 mA, 2Θ range 10–60°) and FTIR spectroscopy (Shimadzu, Kyoto, Japan, IRAffinity1, 400 mg KBr + 1 mg sample).

The microstructures of the cement surfaces were observed using field emission scanning electron microscopy (JEOL FE SEM JSM-7000F, Tokyo, Japan) after coating with carbon. The morphology of particles in the samples was observed using transmission electron microscopy (JEOL JEM 2100F).

The amount of released calcium and phosphorus as a composition of hardened cements (Ca/P ratio) were determined by ICP (Horiba Activa) after their dissolution in HNO_3_ (20%, analytical grade, Merck).

### 2.3. Soaking Cements—pH Measurement, Release of Ca and Phosphates from Cements

The changes in pH during cement soaking at various times (6, 24, 48, 96 and 168 h) were measured using 400 mg cement pellets (6 mm *D* × 12 mm *H*, prepared by molding cement pastes in a stainless-steel mold) hardened for 10 min in 100% humidity and was followed by immersion in 40 mL SBF [26] solutions at 37 °C. The pH of the solution was measured using a pH meter (WTW, Inolab 720, Weilheim, Germany) with a SenTix41 combined electrode. The release of Ca and phosphates was carried out by soaking the cement pellets in 0.9% NaCl solution at 37 °C (350 mg of cement/10 mL of solution). The concentrations of the released elements relative to the solution were analyzed using ICP (Horiba Activa) after 4, 24, 72 and 168 h of soaking.

### 2.4. Measurement of Compressive Strength, Setting Time and Porosity

The compressive strength was measured after molding the cement pastes into cylindrical shapes (6 mm *D* × 12 mm *H*). The samples were soaked in SBF solution at pH = 7.4 and 37 °C for 7 days after setting in 100% humidity at 37 °C for 10 min, which was sufficient time for samples to not disintegrate after immersion in a solution. The compressive strength (mean of 5 samples) of hardened samples was measured on a universal testing machine (5 kN load cell, LR5K Plus, Lloyd Instruments Ltd., West Sussex, UK) at a cross-head speed of 1 mm/min.

The final setting times (ST) of the cement pastes were evaluated using the Vicat method according to ISO standard 1566 [27] but failed to make perceptible circular indentations on the surface of the cement. The porosity of the samples was calculated from the measured dimensions and weight of the samples. The theoretical density of hydroxyapatite (3.15 g/cm^3^) was used for calculation.

### 2.5. Preparation of Cell Extracts and In Vitro Cytotoxicity Testing of Extracts

For in vitro experiments (cytotoxicity, gene expression and Western blotting) rat MSCs isolated from femur and tibia bone marrow were used [28]. The confirmation of the MSC phenotype was verified by immunofluorescence staining of cell-specific markers with conjugated monoclonal antibodies: CD45/CD29/CD90.1 (eBioscience, San Diego, CA, USA) and staining of differentiated cells with commercially purchased kits for the verification of their multi-differentiation capacities (StemPro Osteogenesis, Chondrogenesis and Adipogenesis Differentiation Kit (GIBCO), Waltham, MA, USA) which were used according to manufacturer’s instructions.

The cement extracts were prepared by soaking cement pastes in complete osteogenic differentiation culture medium (α-modification Eagle’s minimum essential medium, EMEM; Biosera, Marikina, Philippines), 10% FBS, osteogenic supplemented with 50 µg/mL of L-ascorbic acid, 50 nM dexamethasone, 10 mM β-glycerophosphate and 1% penicillin, streptomycin, and amphotericin (all Sigma-Aldrich, Saint Louis, MO, USA) in an incubator at 37 °C for 24 h using a ratio of 0.2 g cement powder/mL of medium (M1) (in accordance with ISO 10993-12:2012) [29] or 0.1 g cement/mL medium (M2) (for long-term testing of up to 15 days). Extracts were sterilized by filtration through a membrane (0.2 µm pore size, Millipore, PVDF). MSCs were re-suspended in culture medium after harvesting by enzymatic digestion and cell density was adjusted to 1.0 × 10^5^ cells/mL in a vial. Briefly, 1.0 × 10^4^ of rat MSCs from passage P3 were suspended in 100 µL of EMEM (Biosera) + 10% FBS, 1% antibiotic solution and seeded into each well of a 96-well cell grade Brand microplate (adherent wells) and cultured to a semi-confluent monolayer at 37 °C, 95% humidity, and 5% CO_2_ in an incubator for 24 h. Subsequently, the culture medium in the wells was replaced with 100 µL of 100% extract (M1 or M2). All experiments were carried out in triplicate, and the cells in the wells with extract-free complete culture medium were considered as the negative control. After 24 h of culturing, the M1 extracts were replaced with fresh culture medium and the in vitro cytotoxicity was evaluated (ISO 10993-5:2009 [30]) using the MTS proliferation test assay (cell titer 96 aqueous one solution cell proliferation assay, Promega Madison, WI, USA) using a UV–Vis spectrophotometer (Shimadzu, Kyoto, Japan). M2 extracts were used for long-term MSC viability testing, where culture extracts were changed every two days. The distribution and morphology of the MSCs (10^4^ cells/cement; 6 mm diameter discs) on the surface of hardened CAS and C cements after culturing for 48 h, 7 and 14 days in osteogenic medium were visualized with live/dead staining (fluorescein diacetate/propidium iodide) using an inverted optical fluorescent microscope (Leica DM IL LED, blue filter, Heerbrugg, Switzerland).

Deposits in wells with MSCs cultured in cement extracts were stained with Alizarin red S staining solution for 30 min after washing with PBS, fixing in ethanol for 10 min, and double washing with deionized water. Finally, wells were washed four times with deionized water and observed under a light microscope (Leica DM IL LED).

### 2.6. Gene Expression and SDS PAGE Analysis of Specific Markers

The gene expression was analyzed using a similar method as that in Reference [9]. For the extraction of total RNA, we used approximately 1 × 10^6^ cells. Total RNA from each cell culture was extracted using a RNeasy Mini Kit (Qiagen, Germantown, MD, USA) following the manufacturer’s instructions. Contaminating genomic DNA was digested using an RNase-free DNase set (Qiagen, Germantown, MD, USA). The RNA quality and yields were analyzed using a NanoDrop spectrophotometer (Thermo Scientific, WI, USA). Complementary DNA (cDNA) synthesis was performed using the protocol for the RT2 First Strand Kit (Qiagen, Germantown, MD, USA), where 1 µg of total RNA was used (after the genomic DNA elimination step) to prepare 20 µL of cDNA. cDNA was then used for real time PCR experiments.

The quantification of genes of interest in the cDNA samples was performed using primers for following genes: B-actin rat, type I collagen rat, osteocalcin rat, osteopontin rat, osteonectin rat, and alkaline phosphatase rat (Table 1).

A 25-µL reaction mixture, consisting of triplicate samples of cDNA, specific primer mix, and RT2 SYBR Green qPCR mastermix (Qiagen, Germantown, MD, USA) was setup in each well of a 96-well reaction plate (Roche, Basel, Switzerland). cDNA for β actin was used as the endogenous control for calculating fold differences in the RNA levels of cells treated vs. not treated using biomaterials according to the 2^−ΔΔCT^ method. The plate was sealed using an optical adhesive cover (Roche, Switzerland) and placed in a LightCycler 480 II real time PCR system machine (Roche, Switzerland). Real time PCR was performed under the following conditions: initial incubation at 95 °C for 10 min, amplification in 45 cycles at 95 °C for 15 s followed by 60 °C for 1 min. Amplification specificity was checked by generation of a melting curve.

The cells lysates for SDS PAGE analysis were prepared from cells cultured in M2 extracts, washed in cold PBS, collected and lysed in denaturing cell extraction buffer (Invitrogen, Carlsbad, CA, USA, ThermoFisher, Waltham, MA, USA) supplemented with 1 mM PMSF (phenylmethylsulfonyl fluoride, Sigma-Aldrich, Steinheim, Gernmany) and SIGMAFAST protease inhibitors tablets (Sigma-Aldrich) according to the manufacturer’s instructions. Similar protein amounts (about 20 mg in 30 µL) were run using SDS-PAGE with precasted commercial 10% Bis-Tris mini gels (NuPAGE, Invitrogen) with 1X MES buffer (NuPAGE Novex, Invitrogen). After SDS PAGE, Western blot analysis was carried out using transfer buffer (NuPAGE, Novex, USA) and the semi-dry method (FastBlot semi-dry blotter, Biometra, Germany). Proteins were transferred onto a PVDF membrane (0.45 µm, Invitrolon filter paper sandwich, Invitrogen, Cartsbad, CA, USA) and blocked with skim milk powder (for blotting, SERVA) for 1 h. The membrane was incubated overnight at 4 °C with antibodies against OCN, ON, OP or GAPDH (dilution 1:2000, rabbit anti-rat; Abcam, Cambridge, UK) followed by incubation with secondary antibody goat anti-rabbit IgG HRP conjugated antibody (1:20,000, Invitrogen, Carlsbad, CA, USA). In order to remove non-specific binding, washes with TBS-Tween buffer were performed between each incubation step. Lanes were detected by chemiluminescence after the addition of Pierce ECL Plus Western Blotting Substrate (Pierce Biotechnology, Rockford, IL, USA) and were evaluated using ImageJ software.

## 3. Results and Discussion

### 3.1. XRD and FTIR Analysis of Powder Mixtures and Cements

**Figure 1:**
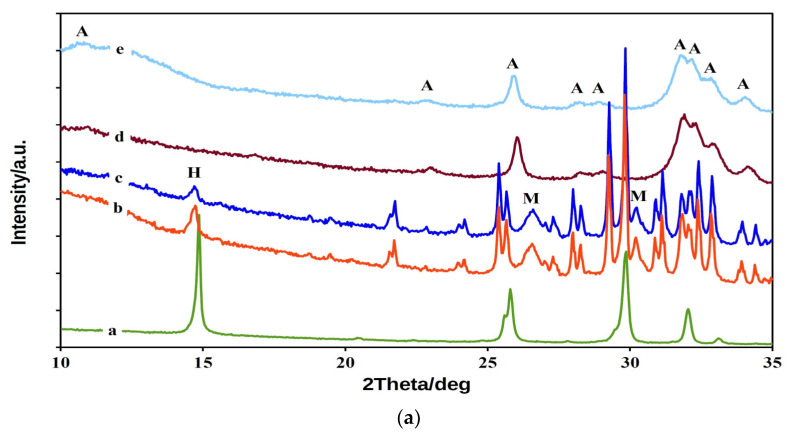

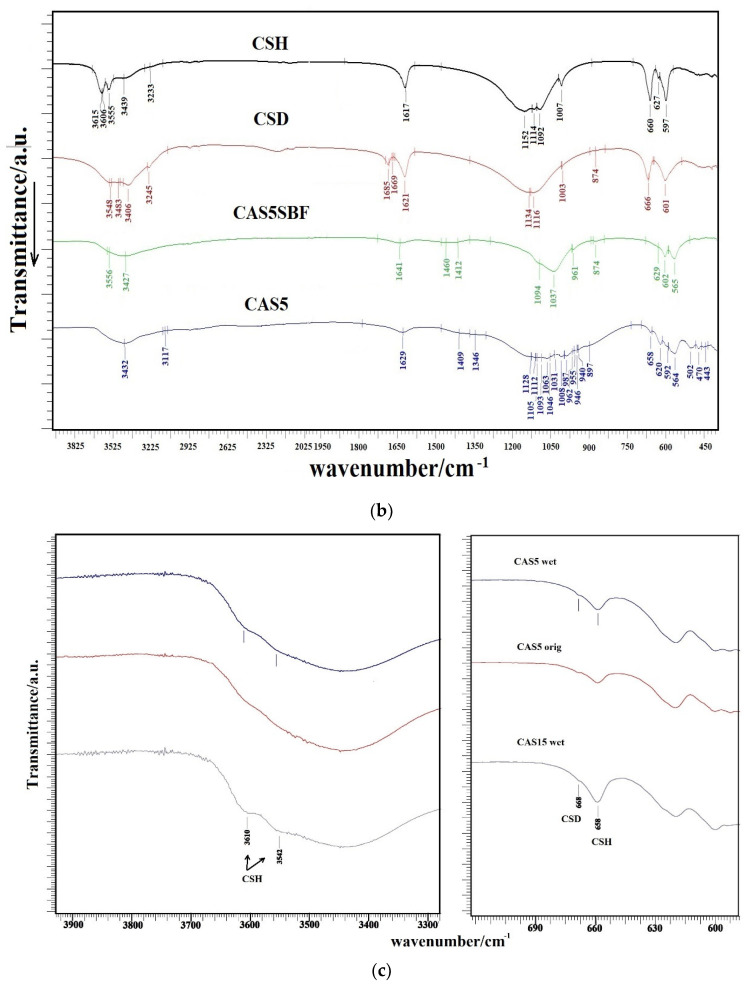
XRD patterns (**a**) (pattern a-CSH standard, pattern b-CAS15 powder mixture, pattern c-CAS5 powder mixture, pattern d-CAS15SBF after soaking in SBF, pattern e-CAS5SBF after soaking in SBF; lines: M-monetite, A-HAP, H-CSH, unsigned lines represent TTCP); FTIR spectra (**b**) of CAS5 cement powder mixture, CAS5SBF after 7 days of soaking at 37 °C in SBF, CSD, CSH standards and (**c**) FTIR spectra of CAS5 and CAS15 after 10 min setting in 100% humidity (spectra CAS5 wet and CAS15 wet respectively) and CAS5 powder cement mixture (spectrum CAS5orig).

The XRD patterns in Figure 1a demonstrate the phase composition of CAS cement powder mixtures and samples after soaking in SBF. A tri-phasic TTCP/monetite/CSH system with patterns of native CAS15 and CAS5 powder mixtures was identified after in situ synthesis. XRD analysis showed the presence of TTCP (JCPDS 25-1137), monetite (JCPDS 09-0080, reflections from (020), (−220) and (−112) planes at 2Θ equal 26.51 and 30.21°) and calcium sulfate hemihydrate (JCPDS 83-0438). The scheme below reflects the chemical reaction taking place during ball milling.

2Ca_4_(PO_4_)_2_O + H_3_PO_4_ + 3H_2_SO_4_ → 3CaSO_4_.1/2H_2_O + 5CaHPO_4_ + 1/2H_2_O (in ball mill)

From comparison of the areas of the lines from reflection of the (002) CSH plane in CAS cement patterns a triple rise in CSH content in CAS15 than CAS5 mixture, which corresponded to the initial preparation conditions, was found. The average CSH particle size in the CAS powder mixtures calculated from the reflection of the (002) CSH plane using the Scherrer equation was about 80 nm. Thus, the applied one-step procedure was suitable for the preparation of TTCPM/nanoCSH cement mixtures without CSD formation. After setting for 7 days in SBF, the full transformation of the starting calcium phosphate phases to nanocrystalline hydroxyapatite (PDF4 01-071-5048), with a minor carbonate substitution, was found in CAS cements. The hydroxyapatite crystallinity sizes calculated from the (002) hydroxyapatite plane were 39 and 32 nm in CAS15 and CAS5 cements, respectively. None of the original CSH or the product of its hydrolysis—CSD—that can form during setting in SBF solution remained, and this was identified in the XRD patterns after soaking in SBF.

In the FTIR spectrum of the starting cement CAS5 powder mixture (Figure 1b), the ν_3_ and ν_1_ stretching vibrations of the ${\mathrm{PO}}_{4}^{3-}$ group at 1063, 1031, 1008 and 987 cm^−1^; ν_4_ and ν_2_ deformation O–P–O vibrations; a symmetric stretching (962–941 cm^−1^) mode (ν_1_) of TTCP [36,37]; shoulders at 1128 and 897 cm^−1^ representing ν_3_ stretching vibrations of P–O and P–O(H) monetite bonds and OH plane bending monetite vibrations at 1409 and 1346 cm^−1^, were found [38]. Furthermore, only low intense CSH peaks (only traces of CSD) may be visible in the spectrum of the original powder mixture due to its low content at 1012, 658 and 1629 cm^−1^, which arise from the bending vibrations of SO_4_(ν_1_), SO_4_(ν_4_) and H_2_O [39]. After 7 days of soaking in SBF, the TTCP and monetite were transformed into nanohydroxyapatite with characteristic vibrations of the ${\mathrm{PO}}_{4}^{3-}$ group—anti symmetric (ν_3_) and symmetric (ν_1_) P–O stretching vibrations at 1037, 1094 and 961 cm^−1^; O–P–O bending (ν_4_) vibrations at 565 and 602 cm^−1^, as well as a weak distinguished liberational mode of the OH group at around 629 cm^−1^, and a peak from stretching vibrations of the OH group at around 3556 cm^−1^. Moreover, bands corresponding to ν_2_ and ν_3_ vibrations of the ${\mathrm{CO}}_{3}^{2-}$ group at 1460, 1412 and 874 cm^−1^ represent the B-type carbonated hydroxyapatite with a ${\mathrm{CO}}_{3}^{2-}$ substitution for ${\mathrm{PO}}_{4}^{3-}$ groups [40]. No peaks from any other phases were identified in the CAS5 spectrum (or CAS15, not shown) after setting.

Note that after 10 min of setting at 100% humidity (Figure 1c), insignificant changes in the composition of CAS5 or CAS15 cement were identified, because, in such a short time, the surface transformation of the starting calcium phosphates with interparticle bond strengthening is possible. The scheme of the transformations of the phase composition of calcium phosphate-based cement after 10 min of setting at 100% humidity, and during soaking in SBF, can be described as follows:

Surface formation of HAP during setting in 100% humidity ↓ CaHPO_4_ + Ca_4_(PO_4_)_2_O (excess in cement) → Ca_5_(PO_4_)_3_OH (soaking) + dissolution of CSH or CSD

It is clear from the present facts that the CAS cement mixtures are highly active in the transformation of the starting phases compared to TTCPM/CSH cements, where the starting TTCP and CSH (or CSD) phases were still detected after 7 days of soaking with TTCPM/55 wt.% CSH cement mixture in Hank’s solution [41], as well as after 6 h of setting of TTCPM/50 wt.% CSD in water [24].

### 3.2. Evaluation of Cement Microstructures—SEM and TEM Analysis

**Figure 2:**
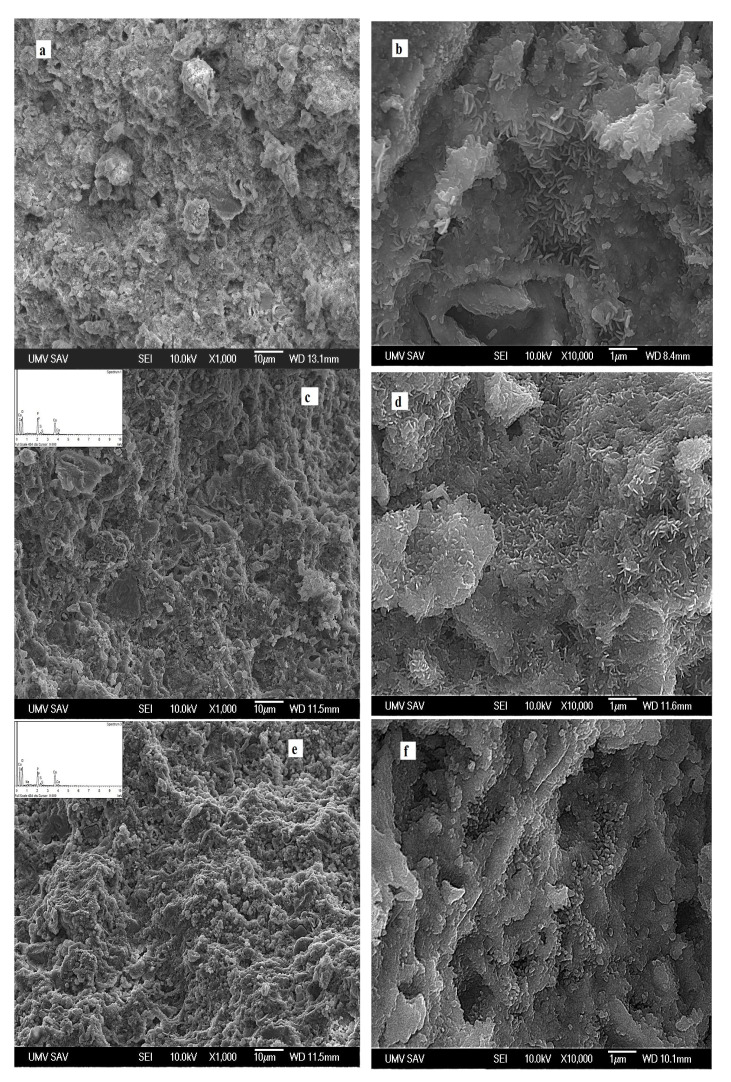

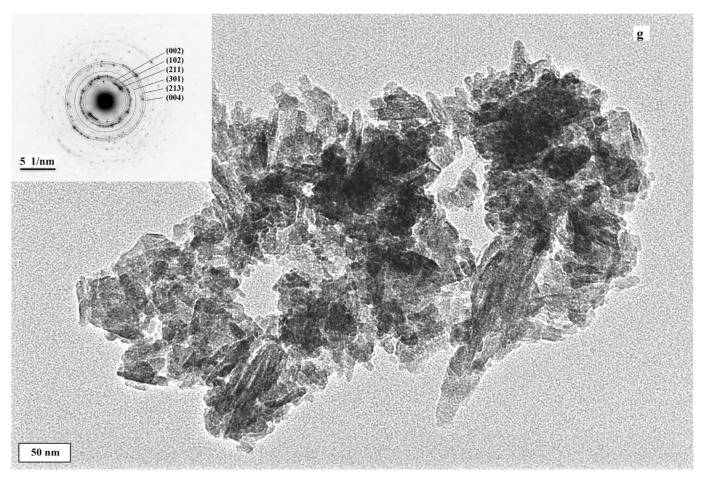
Microstructures of cements after 7 days of setting in SBF: (**a**,**b**) C; (**c**,**d**) CAS15; (**e**,**f**) CAS5; and (**g**) morphology of HAP particles in CAS5 observed using TEM with SAED.

The relatively compact, bigger, and rounded HAP agglomerates, up to 10 µm in size, composed of fine nanometric spherical (<100 nm) or rod-like shaped (length up to 500 nm) particles, can be visible in the microstructure of cement C (without addition of CSH) after setting for 7 days in SBF (Figure 2a,b). In the case of the CAS15 cement microstructure, a lower fraction of irregularly shaped agglomerates, with sizes of 5–10 µm, consisted of thinner, short rod-like HAP nanoparticles (up to 300 nm) surrounded by a very fine matrix was found as compared to C cement (Figure 2c,d). Much finer globular agglomerates (about 1 µm in size) were observed in CAS5 compared to the CAS15 microstructure. Furthermore, the extremely fine spherical or rod-shaped nanoparticles with sizes <100 nm were clearly identified in the CAS5 microstructure (Figure 2e,f). A more detailed TEM analysis (Figure 2g) confirmed the formation of both morphological particle types, and selected area electron diffraction (SAED) verified that they consisted of HAP. Note that area EDX analysis (shown in the SEM images) confirmed the presence of a small amount (<2 wt.%) of sulfur in the CAS5 and CAS15 microstructures. Nevertheless, the calcium sulfate particles could not be individually identified in the microstructure, probably due to the very fine morphology and homogeneous distribution in the cement matrix. In addition, no individual CS particles, or differences in sulfur content between different sites in the microstructure of CAS samples were found using point EDX analysis. This is consistent with the results of XRD or FTIR analysis, in which no CSD was identified. A similar formation of fine plate-like HAP particles in the form of globular agglomerates was observed in CPC/10 wt.% CSH cements after 8 weeks of soaking in SBF [20].

### 3.3. Analysis of Setting Process—Release of Ions, pH Changes, Setting Time and Compressive Strength

C, CAS5 and CAS15 final setting times were 5 ± 1 min (no statistically significant differences, *p* < 0.01) and cements were resistant to washout after setting. The compressive strengths of C, CAS5 and CAS15 cements after 7 days of setting in SBF were 40 ± 4, 31 ± 1.8 and 15 ± 3 MPa, respectively (statistically different, *p* < 0.024). A similar reduction in compressive strength from about 40 MPa to 32 MPa was found in TTCPM and TTCPM/CSH cements (40–60% by weight) due to the dissolution of calcium sulfate, but only a small decrease in ST with CS content (from 10 to 8 min for TTCPM and TTCPM/60 wt.% CSH cements) manifested in the cements [21]. Contrary to above results, a much higher ST (>60 min) was measured in CPC/10 wt.a% CSH composite cement at a P/L ratio = 2.5 [20]. The compressive strength of wet CAS cements was significantly reduced to <5 MPa and to around 11 MPa in CAS15 and CAS5 cements, respectively. Due to the limited use of CAS15 cement for the bone defect treatments, no further evaluation of its properties was performed. The porosities of C and CAS5 cements after hardening in SBF were 52 ± 0.3 and 56 ± 0.6%, respectively (statistically different, *p* < 0.0005). The higher porosity in the CAS5 sample correspond to the dissolution of calcium sulfates during soaking in SBF with a corresponding mass loss in the samples. The influence of CS on the change in cement porosity after an addition of coarse rod-like CSD particles (10–25 wt.%) to αTCP cement showed a porous microstructure with prolonged pores being obtained after dissolving CSD [25]. About 30% porosity was achieved in TTCPM/20 wt.% CSH cements after 7 days of setting in Hank’s solution, but no mention of the L/P ratio is given in [22] despite the fact that it strongly affects the porosity [42]. On the other hand, about 46% porosity was measured in TTCPM cement with P/L = 3 [43], which was a lesser value than that used in C or CAS5 cements due to the lower addition of liquid components. In accordance with our results, rapid mass losses (up to 20 wt.%) of TTCPM cement in TRIS solution were found after 40 and 60 wt.% addition of CSH [21]. Moreover, large amounts of CSH or CSD were identified in αTCP/CSH cement mixtures soaked for 14 days in 0.9% NaCl solution [44] in which results showed that remains of CSD can be present in cements after soaking in solution.

**Figure 3:**
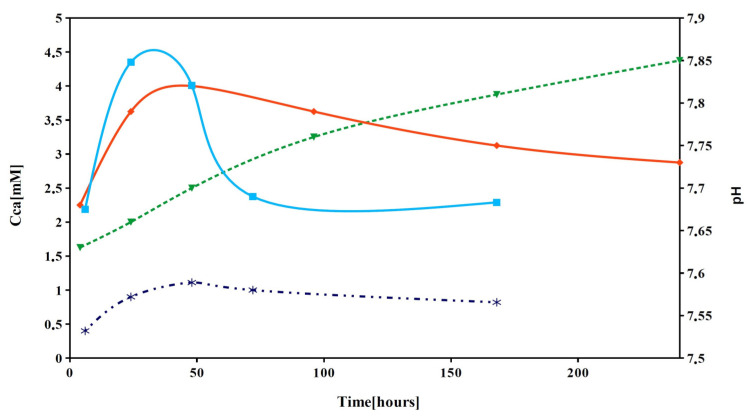
Release of calcium ions (▪CAS5, * C) and pH changes (♦CAS5. V C) during soaking of CAS5 cement at 37 °C in 0.9% NaCl and SBF, respectively.

The changes in the pH of SBF solution during CAS5 cement soaking at 37 °C clearly demonstrated a strong decrease in cement alkalinity, which is characteristic for hydrolysis of calcium phosphate components in TTCPM cements (above 8.5) [45,46], especially shortly after the immersion of cement paste into solution (Figure 3). The maximum pH of SBF (~7.82) was achieved after 48 h of soaking and gradually decreased with soaking time. The pH shift to acidic region (down to 6) was measured during the soaking of TTCPM/20 wt.% CSH cement in Hank’s solution, which confirmed the effect of dissolved calcium sulfates on the fast conversion of cement [22]. Therefore, small variations in pH and ST with CSH content in cements were revealed in SBF solution after immersion of the commercial CPC/CSH cements; compressive strength decreased with CSH content from 45 to 20 MPa for cement free of CSH and 30 wt.% CSH addition (the compressive strength of CPC/10 wt.% CSH was 33 MPa) [20].

Similarly, a rapid rise in Ca concentration was observed after immersion of CAS5 cement in 0.9% NaCl solution at 37 °C for a maximum of 48 h. After 48 h of soaking, the fast decrease in calcium concentration to about half the value of the maximum was recorded. Note that a trace phosphorus concentration, not exceeding 0.1 mM, was measured during soaking. The Ca/P mole ratios in the starting CAS5 powder mixture and 7-day hardened cement were determined by ICP chemical analysis and were 1.75 ± 0.02 and 1.64 ± 0.03, respectively, which corresponds to the theoretical ratio in cement. From the comparison of dependences in Figure 3 four-fold increase of Ca concentration resulted in CAS5 compared to C solution and the pH of C solution gradually rose to 7.85.

A strong influence of CS on setting process of CPC was revealed. The setting time of αTCP/CSD was modulated by the synergy effect of the CSD content in cement in relation to phosphate liquid component where the ST rose with the amount of CSD. This effect was related to the reduction of αTCP hydrolysis after mixing with a liquid component due to the interaction between calcium sulfate and phosphate ions in the liquid [47]. In the case of CAS5 cement, an enhanced concentration of calcium ions occurs during the first stage of setting, which arises from calcium sulfate dissolution, and probably accelerated the transformation of monetite in cement mixture to calcium deficient HAP, which is almost insoluble compared to CSD created from CSH. The acidic character of calcium sulfates promoted by the hydrolysis of TTCP and the released hydroxyl ions are subsequently consumed by the fast interaction between calcium and phosphate ions (from monetite) and HAP precipitation. This fact was supported a low concentration of phosphate ions in the solution during soaking of CAS5 cement. In addition, the precipitation of CSD was decelerated in a later stage of setting process as was recorded by the reduction in Ca concentration after 48 h of cement hardening because CSD solubility is half that of CSH, despite the higher thermodynamic stability of the CSD phase [48]. The strong suppression of both the nucleation and growth of CSD, which is created from CSH, was confirmed by calorimetric measurements during the transformation of TTCPM/CSH composites, even at 10 wt.% addition of TTCPM to CSH. Similar results were shown after soaking αTCP/CSH mixtures in NaCl solution, where the amount of untransformed CSH rose with the content of αTCP in cement [44]. All presented facts showed that dissolved CS actively influenced the CAS5 cement setting process.

### 3.4. In Vitro Testing—Evaluation of Cytotoxicity Cement Extracts, Gene Expression of Specific Markers

The cells isolated from rat bone marrow at passage 3 were positive stained for cartilage formation with Alcian blue, calcium deposit formation with Alizarin red, and adipocyte formation with oil red. Only 1.1% of tested cells expressed CD45 markers and 95.5% were CD29+CD90.1+ according to flow cytometry. The isolated cells belonged to the MSC phenotype and were used for in vitro experiments.

**Figure 4:**
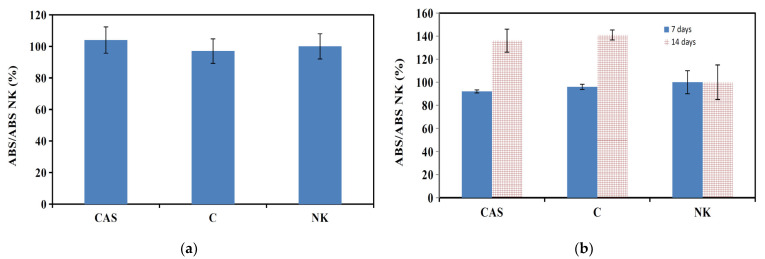
Viability of osteoblasts cultured in negative control (NK), CAS5 and C cement M1 extracts for 24 h (**a**) and M2 extracts for 7 and 14 days (**b**).

**Figure 5:**
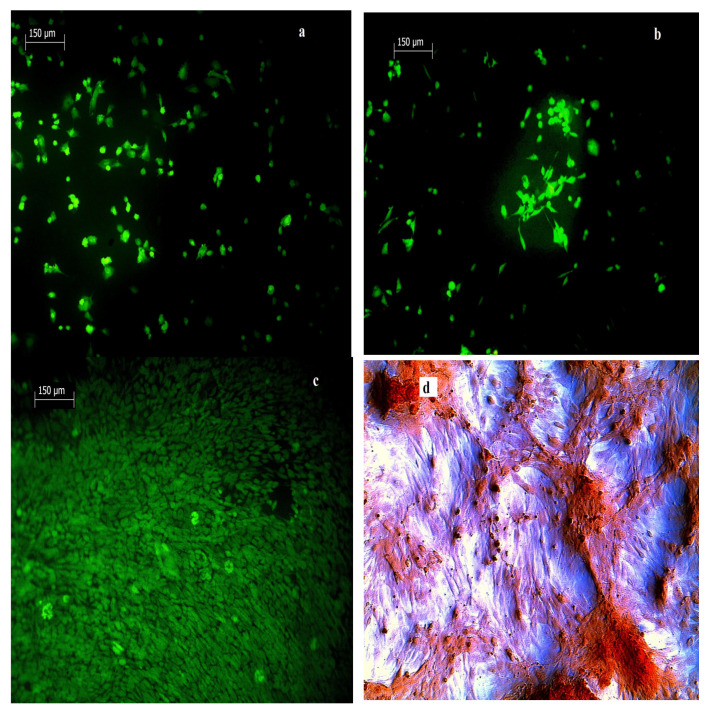

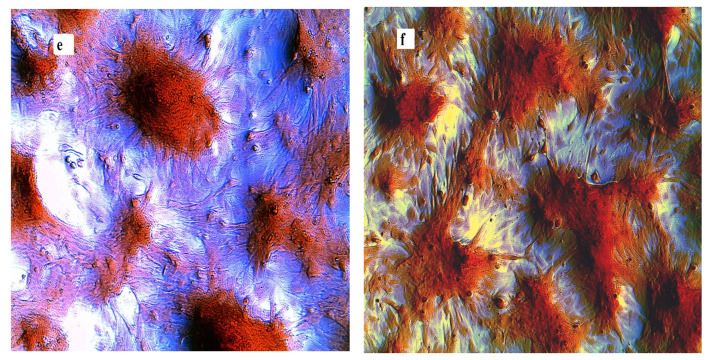
Morphology and distribution of cells cultured on CAS5 cement for 48 h (**a**), 7 (**b**) and 14 (**c**) days (live/dead staining) and production of calcium deposits by cells cultured in negative control (**d**), C (**e**) and CAS5 (**f**) M2 extracts for 10 days.

The evaluation of in vitro cytotoxicity of cement extracts (M1) (ISO 10993-5:2009) using rat MSCs showed their non-cytotoxic character (Figure 4a). Similarly, the long-term cultivation of MSCs in cement extracts (M2) verified a good viability and even enhanced proliferation of MSCs after 14 days of culture in both C and CAS5 cement extracts (Figure 4b). In Figure 5a–c, the morphology and density of MSCs cultured at various times on the surface of CAS5 cement are shown. A good adherence and cell spread after 48 h of culture in osteogenic medium was observed.

After 7 days of culturing, the large fraction of cells had spindle-like shape with the long filopodia, which is characteristic for osteoblast-like cells, many cells were well spread and had a much higher contact area with the surface of CAS5 cement, which demonstrates the non-cytotoxic character of the cement surface and its appropriate adherence (Figure 5b). The number of cells grew rapidly during 14 days of culture (Figure 5c) and a multilayered cell coating was found on the cement surface. The ability of cells to produce calcium deposits in C and CAS5 cement extracts was identified using Alizarin red staining of deposits (Figure 5d–f). From a comparison of the images we found that the calcium deposits were produced in wells with cells cultured in M2 cement extracts, as well as in the negative control (medium free of cements), but the content of deposits was clearly higher in wells with cement extracts, and the CAS5 extract significantly supported differentiation of MSCs and their ability to perform calcification. Non-cytotoxic character of the extract from the TTCPM cement mixture containing even 55 wt.% of CSH, as well as enhanced remodeling activities with new bone network development within a femoral defect in rabbit was identified [41].

**Figure 6:**
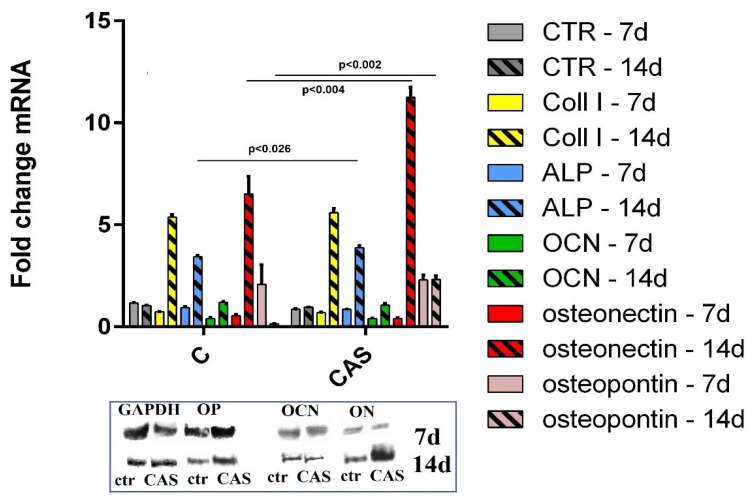
Relative gene expression of COL1, OCN, ON, OP and ALP in MSCs cultured for 7 and 14 days in M2 extracts (statistically significant differences between ctr and C (probability 7/14 days)): COLL1 (<0.008/<0.0001); ALP (<0.05/<0.001); OCN (<0.003/not different (ND)) >0.09; ON (<0.0001/<0.0001); OP (ND > 0.091/<0.0001; statistically significant differences between ctr and CAS5 (probability 7/14 days)): COLL1 (<0.008/<0.0001); ALP (<0.05/<0.001); OCN (<0.002/not different (ND)) >0.09; ON (<0.0001/<0.0001); OP (<0.0004/<0.0004) and SDS PAGE analysis of lysates from MSCs cultured in M2 extracts for 7 and 14 days (detail in frame).

For documentation of the osteoblastic activity of differentiated MSCs, the osteogenic markers and osteogenic gene expressions for ALP, OCN, OP, ON and COLL I were evaluated using SDS-PAGE and RT-qPCR analysis. The relative gene expression (Figure 6) identified a statistically significant down regulation of COLL I, ALP, OCN, ON and over expression of OP in MSCs cultured for 7 days in C and CAS5 cement extracts compared to the control sample. The over expression of COLL 1, ALP, and ON genes was found in MSCs after long-term cultivation of up to 14 days in both cement extracts, whereas OP was down-regulated and up-regulated in cells cultured in C and CAS5 extracts, respectively. RT-qPCR findings verified the results from SDS PAGE analysis (details in Figure 6). The comparison between gene expression in cells of C and CAS5 groups revealed statistically significantly higher gene expression of ALP, ON and OP in CAS5 than C cement extracts. It is known that osteopontin in a mixture with osteocalcin, promotes hydroxyapatite formation [49]. Osteonectin is a tissue-specific protein that helps to link bone mineral and collagen phases and is expressed at high levels in active osteoblasts [50,51]. Both markers and ALP are induced in the mineralization stage of osteoblasts. The significant enhancement of ALP activity and up-regulation of OC, ON and COLL 1 genes were found in cells seeded on HAP substrates at only 5% sulfate substitution of phosphate ions [12], which verifies the stimulation effect of increased HAP solubility on osteogenic activity of cells. Moreover, it was demonstrated that the proliferation of osteoblasts was enhanced at higher concentrations of Ca^2+^ ions (2–6 mM) and further increases (up to 10 mM) improved the differentiation of osteoblasts and matrix mineralization [52]. On the other hand, calcium sulfate addition to culture medium in a very small concentration (0.001 mg/mL) had a significant effect on the differentiation of dental pulp stem cells, with up-regulation of OP, ALP and COLL 1 gene expression after 15 days of culture [53]. The 3–5 mM CS addition to culture medium promoted in vitro MSC migration in a wound healing assay and caused the down regulation of ALP and OCN gene expression during first 4 days of culture followed by over expression of both genes [54]. This fact verified the promoting of cell differentiation at low CS concentrations, which is in an accordance with our results. The addition of homogeneously distributed nanoCSH to TTCPM cement mixtures, even in a relatively small amount (around 5 wt.%), significantly improved the osteogenic bioactivity of the composite cement in relation to the differentiation of MSCs, which is a prerequisite for enhanced new in vivo bone formation during bone defect treatments.

## 4. Conclusions

TTCP/monetite/CSH composite cements were prepared using a modified one-step in situ preparation procedure, where powdered tetracalcium phosphate was milled with an ethanolic solution of both orthophosphoric and sulfuric acids. Monetite and CSH were present in final powder cement mixtures in the form of homogeneously distributed nanoparticles. No changes in setting time (around 5 min) were measured after addition of CSH in 5 and 15 wt.% to the TTCP/monetite cement mixture. Compressive strength decreased with CSH content and it was half (of 15 MPa) after the addition of 15 wt.% CSH to cement. It was demonstrated that the fast release of calcium ions from soluble CSH to physiological solution and effective suppression of the starting pH increase in CAS cement pastes. The non-cytotoxic character of the cement surface verified a high fraction of well-spread live cells with good adherence to CAS5 cement after 14 days of culture. The RT-PCR and SDS PAGE analyses confirmed significantly enhanced osteogenic activity of MSCs cultured in extract from CAS5 cement (with 5 wt.% of CSH only) as compared with extract from cement free of CSH.

## Figures and Tables

**Table 1:** Forward and reverse primers of genes used for RT-PCR experiments.

Genes	Primers (5′3′)	Reference
B-actin rat	F: GTAGCCATCCAGGCTGTGTT R: CCCTCATAGATGGGCAGAGT	[31]
Type I collagen rat	F: CCAGCTGACCTTCCTGCGCC R: CGGTGTGACTCGTGCAGCCA	[32]
Osteocalcin rat	FACAGACAAGTCCCACACAGCAACT R: CCTGCTTGGACATGAAGGCTTTGT	[33]
Osteopontin rat	F: CCGATGAATCTGATGAGTCCTT R: TCCAGCTGACTTGACTCATGG	[34]
Osteonectin rat	F: GGAAGCTGCAGAAGAGATGG R: TGCACACCTTTTCAAACTCG	[34]
Alkaline phosphatase rat	F: AACCTGACTGACCCTTCCCTCT R: TCAATCCTGCCTCCTTCCACTA	[35]

## Data Availability

Data Sharing is not applicable.
